# Pharmacological Therapies for Pain Management in Patients With Amputation: A Systematic Review and Network Meta‐Analysis

**DOI:** 10.1155/prm/3654470

**Published:** 2026-06-04

**Authors:** Nahadja Tahaynara Barros Leal, Larissa Régia da Fonseca Marinho, Vinicius dos Santos Lemos Pereira, Bruna Carmelita Rocha Pontes, Ayane Cristine Alves Sarmento, Kleyton Santos de Medeiros, Katia Regina Barros Ribeiro, Rodrigo Assis Neves Dantas, Daniele Vieira Dantas

**Affiliations:** ^1^ Graduate Program in Nursing, Federal University of Rio Grande do Norte, Natal, Rio Grande do Norte, Brazil, ufrn.br; ^2^ Graduate in Nursing, Federal University of Rio Grande do Norte, Natal, Rio Grande do Norte, Brazil, ufrn.br; ^3^ Institute of Education, Research, and Innovation, Liga Contra o Câncer, Natal, Rio Grande do Norte, Brazil; ^4^ Graduate Program in Health Sciences, Federal University of Rio Grande do Norte, Natal, Rio Grande do Norte, Brazil, ufrn.br; ^5^ Department of Nursing, Federal University of Rio Grande do Norte (UFRN), Natal, Rio Grande do Norte, Brazil, ufrn.br

**Keywords:** amputation, drug therapy, pain, pain management

## Abstract

**Objective:**

To evaluate the efficacy and safety of pharmacological therapies for pain management in postamputation patients through a systematic review and network meta‐analysis of existing evidence.

**Methods:**

This systematic review and network meta‐analysis (NMA) was conducted following the PRISMA‐NMA guidelines and registered in PROSPERO (CRD42024500600). In September 2024, two independent reviewers searched the Cochrane Library, LILACS, PubMed/MEDLINE, SciELO, ScienceDirect, Web of Science, Scopus, CINAHL, and Google Scholar. The eligibility criteria included systematic reviews (with or without meta‐analysis) evaluating pharmacological interventions in populations aged 18 years or older.

**Results:**

Seventeen studies met the inclusion criteria and were described in narrative form. Among these studies, seven systematic reviews of randomized clinical trials contained data suitable for NMA. Two primary outcomes were assessed: residual limb pain and phantom limb pain. Analysis of the three subnetworks revealed no statistically significant differences in either direct or indirect comparisons across the evaluated therapies.

**Conclusion:**

Current evidence is insufficient to support the efficacy and safety of specific pharmacological therapies for pain management in patients with amputation compared to placebo or alternative treatments.

## 1. Introduction

Amputation is a profound physical and psychological trauma with a high global incidence. Beyond the loss of mobility, patients frequently face persistent pain, increased dependency, diminished quality of life, and a higher prevalence of depression and other mental health disorders [1].

Pain remains the most prevalent clinical symptom in the postoperative period following amputation [2]. Most patients experience two distinct manifestations: residual limb pain (RLP), which typically subsides after tissue healing, and phantom limb pain (PLP), which persists beyond the healing phase and can become a lifelong condition [3].

Effective management requires the healthcare team to periodically assess and differentiate these clinical forms. Prompt and individualized treatment is essential to prevent the transition from acute to chronic pain, thereby promoting patient well‐being and functional recovery [1, 4]. Current strategies to alleviate post‐amputation pain include specialized surgical techniques, regional analgesia, pharmacological interventions, psychotherapy, and supportive care such as stump dressings, cryotherapy, therapeutic massage, mirror therapy, and patient education regarding prosthesis care [5].

Despite this multidisciplinary repertoire, a consensus on the most effective pain control protocol remains elusive in the scientific literature, partly due to a pathophysiology that is not yet fully understood [5–10]. Commonly utilized pharmacological therapies include opioid and nonopioid analgesics, nonsteroidal anti‐inflammatory drugs (NSAIDs), epidural or perineural anesthetics, N‐methyl‐D‐aspartate (NMDA) receptor antagonists, gabapentinoids, antidepressants, and calcitonin [2, 5, 9].

However, the evidence regarding the efficacy of these treatments is often divergent, and the wide range of associated adverse effects poses significant risks to patient safety [1, 2, 5, 9, 11]. Furthermore, there is a lack of high‐level evidence synthesis that meticulously compares these therapies to establish their comparative short‐ and long‐term benefits.

Given that postamputation pain causes substantial suffering and that medications are often the primary intervention in clinical settings, bridging these literature gaps is critical. Since the appropriate selection of therapy directly impacts symptom control and clinical outcomes, this study seeks to provide a robust synthesis of the existing evidence [10–12].

The aim of this study is to evaluate the efficacy and safety of pharmacological therapies for pain management in patients undergoing amputation. By conducting a comprehensive systematic review, this research seeks to support evidence‐based therapeutic choices, stimulate academic debate, and generate knowledge that clarifies the clinical impact of these interventions. Furthermore, the inclusion of a network meta‐analysis (NMA) allows for the comparative ranking of treatments, providing a hierarchical evidence base to support clinical decision‐making and optimize patient care.

## 2. Materials and Methods

This study consists of a systematic review and NMA conducted in accordance with the Cochrane Handbook for Systematic Reviews of Interventions [13]. The reporting follows the Preferred Reporting Items for Systematic Reviews and Meta‐Analyses for NMA (PRISMA‐NMA) statement [14] (Supporting File 1). The study protocol was prospectively registered in the PROSPERO database under the identifier CRD42024500600 (Supporting File 2).

### 2.1. Search Strategy

To develop the search strategy, relevant descriptors were identified and selected based on the PICOS framework, defined as follows:

• Population (P): adults and older adults (aged ≥ 18 years) with limb amputation.
• Intervention (I): pharmacological therapies.
• Control (C): placebo or alternative therapies.
• Outcomes (O): pain management.
• Study design (S): systematic reviews with or without meta‐analysis.

Search terms were selected according to the Medical Subject Headings (MeSH) and Health Sciences Descriptors (DeCS). Boolean operators (AND, OR) were applied to combine descriptors into specific strings optimized for each database. Preliminary searches were conducted across all databases to refine these combinations according to the specific indexing features of each source, as per Table 1.

**TABLE 1:** Search syntax adapted for each data source. Natal, RN, Brazil, 2025.

Data source	Search syntax
Cochrane Library	“Amputation” OR “Amputation surgical” OR “Physiologic amputation” OR “Post amputation syndrome” OR “Reamputation” OR “Traumatic amputation” OR “Amputation, traumatic” OR “Multiple amputations, traumatic” OR “Multiple traumatic amputations” OR “Multiple traumatic limb amputations” OR “Traumatic amputation” OR “Amputation stump” OR “Amputation stump” OR “Amputation stumps” OR “Exarticulation stump” OR “Stump, amputation” in Title Abstract Keyword AND “Analgesia” OR “Pain management” OR “Pain relief” OR “Sequential analgetic analgesia” OR “Surgical analgesia” OR “Drug therapy” OR “Drug treatment” OR “Medicament therapy” OR “Medicament treatment” OR “Medication” OR “Medicinal therapy” OR “Medicinal treatment” OR “Pharmaceutical therapy” OR “Pharmaceutical treatment” OR “Pharmaco‐therapy” OR “Pharmaco‐treatment” OR “Pharmacological therapy” OR “Pharmacological treatment” OR “Pharmacotherapy” OR “Pharmacotreatment” OR “Therapeutic uses” OR “Therapy, drug” OR “Therapy, pharmacological” OR “Treatment, drug” OR “Treatment, pharmacological” in Title Abstract Keyword AND “Pain” OR “Acute pain” OR “Deep pain” OR “Lightning pain” OR “Nocturnal pain” OR “Pain” OR “Pain response” OR “Pain syndrome” OR “Treatment related pain” OR “Chronic pain” OR “Chronic intractable pain” OR “Chronic persistent pain” OR “Phantom limb” OR “Phantom limb sensation” OR “Phantom limb syndrome” OR “Phantom sensation” OR “Pseudomelia” in Title Abstract Keyword AND “Systematic review” OR “Review” OR “Meta analysis” OR “Meta‐analysis” OR “Metaanalysis” in Title Abstract Keyword.
LILACS	(“Amputation” OR “Amputation surgical” OR “Physiologic amputation” OR “Post amputation syndrome” OR “Reamputation” OR “Traumatic amputation” OR “Amputation, traumatic” OR “Multiple amputations, traumatic” OR “Multiple traumatic amputations” OR “Multiple traumatic limb amputations” OR “Traumatic amputation” OR “Amputation stump” OR “Amputation stump” OR “Amputation stumps” OR “Exarticulation stump” OR “Stump, amputation”) AND (“Analgesia” OR “Pain management” OR “Pain relief” OR “Sequential analgetic analgesia” OR “Surgical analgesia” OR “Drug therapy” OR “Drug treatment” OR “Medicament therapy” OR “Medicament treatment” OR “Medication” OR “Medicinal therapy” OR “Medicinal treatment” OR “Pharmaceutical therapy” OR “Pharmaceutical treatment” OR “Pharmaco‐therapy” OR “Pharmaco‐treatment” OR “Pharmacological therapy” OR “Pharmacological treatment” OR “Pharmacotherapy” OR “Pharmacotreatment” OR “Therapeutic uses” OR “Therapy, drug” OR “Therapy, pharmacological” OR “Treatment, drug” OR “Treatment, pharmacological”) AND (“Pain” OR “Acute pain” OR “Deep pain” OR “Lightning pain” OR “Nocturnal pain” OR “Pain” OR “Pain response” OR “Pain syndrome” OR “Treatment related pain” OR “Chronic pain” OR “Chronic intractable pain” OR “Chronic persistent pain” OR “Phantom limb” OR “Phantom limb sensation” OR “Phantom limb syndrome” OR “Phantom sensation” OR “Pseudomelia”) AND (“Systematic review” OR “Review” OR “Meta analysis” OR “Meta‐analysis” OR “Metaanalysis”).
PubMed	(‘amputation’/exp OR ‘amputation’ OR ‘amputation, surgical’ OR ‘physiologic amputation’ OR ‘post amputation syndrome’ OR ‘reamputation’ OR ‘traumatic amputation’/exp OR ‘amputation, traumatic’ OR ‘multiple amputations, traumatic’ OR ‘multiple traumatic amputations’ OR ‘multiple traumatic limb amputations’ OR ‘traumatic amputation’ OR ‘amputation stump’/exp OR ‘amputation stump’ OR ‘amputation stumps’ OR ‘exarticulation stump’ OR ‘stump, amputation’) AND (‘analgesia’/exp OR ‘analgaesia’ OR ‘analgesia’ OR ‘pain management’ OR ‘pain relief’ OR ‘sequential analgetic analgesia’ OR ‘surgical analgesia’ OR ‘drug therapy’/exp OR ‘drug therapy’ OR ‘drug treatment’ OR ‘medicament therapy’ OR ‘medicament treatment’ OR ‘medication’ OR ‘medicinal therapy’ OR ‘medicinal treatment’ OR ‘pharmaceutical therapy’ OR ‘pharmaceutical treatment’ OR ‘pharmaco‐therapy’ OR ‘pharmaco‐treatment’ OR ‘pharmacological therapy’ OR ‘pharmacological treatment’ OR ‘pharmacotherapy’ OR ‘pharmacotreatment’ OR ‘therapeutic uses’ OR ‘therapy, drug’ OR ‘therapy, pharmacological’ OR ‘treatment, drug’ OR ‘treatment, pharmacological’) AND (‘pain′/exp OR ‘acute pain’ OR ‘deep pain’ OR ‘lightning pain’ OR ‘nocturnal pain’ OR ‘pain’ OR ‘pain response’ OR ‘pain syndrome’ OR ‘treatment related pain’ OR ‘chronic pain’/exp OR ‘chronic intractable pain’ OR ‘chronic pain’ OR ‘chronic persistent pain’ OR ‘pain, chronic’ OR ‘phantom limb’/exp OR ‘phantom limb’ OR ‘phantom limb sensation’ OR ‘phantom limb syndrome’ OR ‘phantom sensation’ OR ‘pseudomelia’) AND (‘systematic review’/exp OR ‘review, systematic’ OR ‘systematic review’ OR ‘review’/exp OR ‘review’ OR ‘meta analysis’/exp OR ‘analysis, meta’ OR ‘meta analysis’ OR ‘meta‐analysis’ OR ‘metaanalysis’).
SciELO	(“Amputation” OR “Amputation surgical” OR “Physiologic amputation” OR “Post amputation syndrome” OR “Reamputation” OR “Traumatic amputation” OR “Amputation, traumatic” OR “Multiple amputations, traumatic” OR “Multiple traumatic amputations” OR “Multiple traumatic limb amputations” OR “Traumatic amputation” OR “Amputation stump” OR “Amputation stump” OR “Amputation stumps” OR “Exarticulation stump” OR “Stump, amputation”) AND (“Analgesia” OR “Pain management” OR “Pain relief” OR “Sequential analgetic analgesia” OR “Surgical analgesia” OR “Drug therapy” OR “Drug treatment” OR “Medicament therapy” OR “Medicament treatment” OR “Medication” OR “Medicinal therapy” OR “Medicinal treatment” OR “Pharmaceutical therapy” OR “Pharmaceutical treatment” OR “Pharmaco‐therapy” OR “Pharmaco‐treatment” OR “Pharmacological therapy” OR “Pharmacological treatment” OR “Pharmacotherapy” OR “Pharmacotreatment” OR “Therapeutic uses” OR “Therapy, drug” OR “Therapy, pharmacological” OR “Treatment, drug” OR “Treatment, pharmacological”) AND (“Pain” OR “Acute pain” OR “Deep pain” OR “Lightning pain” OR “Nocturnal pain” OR “Pain” OR “Pain response” OR “Pain syndrome” OR “Treatment related pain” OR “Chronic pain” OR “Chronic intractable pain” OR “Chronic persistent pain” OR “Phantom limb” OR “Phantom limb sensation” OR “Phantom limb syndrome” OR “Phantom sensation” OR “Pseudomelia”) AND (“Systematic review” OR “Review” OR “Meta analysis” OR “Meta‐analysis” OR “Metaanalysis”).
ScienceDirect	(“Amputation”) AND (“Analgesia” OR “Pain relief” OR “Drug therapy”) AND (“Pain” OR “Phantom limb” OR “Phantom limb sensation”) AND (“Systematic review”).
Scopus	(“Amputation” OR “Amputation surgical” OR “Physiologic amputation” OR “Post amputation syndrome” OR “Reamputation” OR “Traumatic amputation” OR “Amputation, traumatic” OR “Multiple amputations, traumatic” OR “Multiple traumatic amputations” OR “Multiple traumatic limb amputations” OR “Traumatic amputation” OR “Amputation stump” OR “Amputation stump” OR “Amputation stumps” OR “Exarticulation stump” OR “Stump, amputation”) AND (“Analgesia” OR “Pain management” OR “Pain relief” OR “Sequential analgetic analgesia” OR “Surgical analgesia” OR “Drug therapy” OR “Drug treatment” OR “Medicament therapy” OR “Medicament treatment” OR “Medication” OR “Medicinal therapy” OR “Medicinal treatment” OR “Pharmaceutical therapy” OR “Pharmaceutical treatment” OR “Pharmaco‐therapy” OR “Pharmaco‐treatment” OR “Pharmacological therapy” OR “Pharmacological treatment” OR “Pharmacotherapy” OR “Pharmacotreatment” OR “Therapeutic uses” OR “Therapy, drug” OR “Therapy, pharmacological” OR “Treatment, drug” OR “Treatment, pharmacological”) AND (“Pain” OR “Acute pain” OR “Deep pain” OR “Lightning pain” OR “Nocturnal pain” OR “Pain” OR “Pain response” OR “Pain syndrome” OR “Treatment related pain” OR “Chronic pain” OR “Chronic intractable pain” OR “Chronic persistent pain” OR “Phantom limb” OR “Phantom limb sensation” OR “Phantom limb syndrome” OR “Phantom sensation” OR “Pseudomelia”) AND (“Systematic review” OR “Review” OR “Meta analysis” OR “Meta‐analysis” OR “Metaanalysis”).
CINAHL	(“Amputation” OR “Amputation surgical” OR “Physiologic amputation” OR “Post amputation syndrome” OR “recomputation” OR “Traumatic amputation” OR “Amputation, traumatic” OR “Multiple amputations, traumatic” OR “Multiple traumatic amputations” OR “Multiple traumatic limb amputations” OR “Traumatic amputation” OR “Amputation stump” OR “Amputation stump” OR “Amputation stumps” OR “Exarticulation stump” OR “Stump, amputation”) AND (“Analgesia” OR “Pain management” OR “Pain relief” OR “Sequential analgetic analgesia” OR “Surgical analgesia” OR “Drug therapy” OR “Drug treatment” OR “Medicament therapy” OR “Medicament treatment” OR “Medication” OR “Medicinal therapy” OR “Medicinal treatment” OR “Pharmaceutical therapy” OR “Pharmaceutical treatment” OR “Pharmaco‐therapy” OR “Pharmaco‐treatment” OR “Pharmacological therapy” OR “Pharmacological treatment” OR “Pharmacotherapy” OR “pharmacotreatments” OR “Therapeutic uses” OR “Therapy, drug” OR “Therapy, pharmacological” OR “Treatment, drug” OR “Treatment, pharmacological”) AND (“Pain” OR “Acute pain” OR “Deep pain” OR “Lightning pain” OR “Nocturnal pain” OR “Pain” OR “Pain response” OR “Pain syndrome” OR “Treatment related pain” OR “Chronic pain” OR “Chronic intractable pain” OR “Chronic persistent pain” OR “Phantom limb” OR “Phantom limb sensation” OR “Phantom limb syndrome” OR “Phantom sensation” OR “pseudovelia”) AND (“Systematic review” OR “Review” OR “Meta analysis” OR “Meta‐analysis” OR “Metaanalysis”).
Web of Science	“Amputation” OR “Amputation surgical” OR “Physiologic amputation” OR “Post amputation syndrome” OR “recomputation” OR “Traumatic amputation” OR “Amputation, traumatic” OR “Multiple amputations, traumatic” OR “Multiple traumatic amputations” OR “Multiple traumatic limb amputations” OR “Traumatic amputation” OR “Amputation stump” OR “Amputation stump” OR “Amputation stumps” OR “Exarticulation stump” OR “Stump, amputation” (All Fields) and “Analgesia” OR “Pain management” OR “Pain relief” OR “Sequential analgetic analgesia” OR “Surgical analgesia” OR “Drug therapy” OR “Drug treatment” OR “Medicament therapy” OR “Medicament treatment” OR “Medication” OR “Medicinal therapy” OR “Medicinal treatment” OR “Pharmaceutical therapy” OR “Pharmaceutical treatment” OR “Pharmaco‐therapy” OR “Pharmaco‐treatment” OR “Pharmacological therapy” OR “Pharmacological treatment” OR “Pharmacotherapy” OR “pharmacotreatments” OR “Therapeutic uses” OR “Therapy, drug” OR “Therapy, pharmacological” OR “Treatment, drug” OR “Treatment, pharmacological” (All Fields) and “Pain” OR “Acute pain” OR “Deep pain” OR “Lightning pain” OR “Nocturnal pain” OR “Pain” OR “Pain response” OR “Pain syndrome” OR “Treatment related pain” OR “Chronic pain” OR “Chronic intractable pain” OR “Chronic persistent pain” OR “Phantom limb” OR “Phantom limb sensation” OR “Phantom limb syndrome” OR “Phantom sensation” OR “pseudovelia” (All Fields) and “Systematic review” OR “Review” OR “Meta analysis” OR “Meta‐analysis” OR “Metaanalysis” (All Fields).
Google Acadêmico	(“Amputation” OR “Amputation surgical” OR “Physiologic amputation” OR “Post amputation syndrome” OR “Reamputation” OR “Traumatic amputation” OR “Amputation, traumatic” OR “Multiple amputations, traumatic” OR “Multiple traumatic amputations” OR “Multiple traumatic limb amputations” OR “Traumatic amputation” OR “Amputation stump” OR “Amputation stump” OR “Amputation stumps” OR “Exarticulation stump” OR “Stump, amputation”) AND (“Analgesia” OR “Pain management” OR “Pain relief” OR “Sequential analgetic analgesia” OR “Surgical analgesia” OR “Drug therapy” OR “Drug treatment” OR “Medicament therapy” OR “Medicament treatment” OR “Medication” OR “Medicinal therapy” OR “Medicinal treatment” OR “Pharmaceutical therapy” OR “Pharmaceutical treatment” OR “Pharmaco‐therapy” OR “Pharmaco‐treatment” OR “Pharmacological therapy” OR “Pharmacological treatment” OR “Pharmacotherapy” OR “Pharmacotreatment” OR “Therapeutic uses” OR “Therapy, drug” OR “Therapy, pharmacological” OR “Treatment, drug” OR “Treatment, pharmacological”) AND (“Pain” OR “Acute pain” OR “Deep pain” OR “Lightning pain” OR “Nocturnal pain” OR “Pain” OR “Pain response” OR “Pain syndrome” OR “Treatment related pain” OR “Chronic pain” OR “Chronic intractable pain” OR “Chronic persistent pain” OR “Phantom limb” OR “Phantom limb sensation” OR “Phantom limb syndrome” OR “Phantom sensation” OR “Pseudomelia”) AND (“Systematic review” OR “Review” OR “Meta analysis” OR “Meta‐analysis” OR “Metaanalysis”).

*Note:* Source: Research data, 2025.

Following the finalization of the search strings, the systematic search was performed by two independent, calibrated researchers to ensure methodological rigor.

### 2.2. Eligibility Criteria

We included systematic reviews, with or without meta‐analysis, evaluating pharmacological interventions for pain management in patients aged 18 years or older (adults and older adults) who had undergone amputation. No restrictions were placed on publication date or language, provided the full text was available.

Studies were excluded if they (1) were duplicates; (2) did not directly address the research question; (3) and were unavailable in full‐text format or had restricted access. Furthermore, gray literature including editorials, manuals, dissertations, theses, study protocols, pilot studies, conference abstracts, and advertisements was excluded to ensure the inclusion of peer‐reviewed scientific research only.

### 2.3. Search Strategy and Study Selection

The search was conducted in September 2024 across the following databases: Cochrane Library, LILACS, PubMed/MEDLINE, SciELO, ScienceDirect, Scopus, CINAHL, Web of Science, and Google Scholar. Most data sources were accessed through the Coordination for the Improvement of Higher Education Personnel (CAPES) Periodicals Portal via the Federated Academic Community (CAFe), facilitated by the Federal University of Rio Grande do Norte (UFRN). The Cochrane Library and Google Scholar were accessed directly through their respective official platforms.

To ensure methodological rigor and minimize the risk of bias, errors, or discrepancies, all stages of the review including the search, study selection, and data extraction were performed by two independent, calibrated researchers.

The Rayyan systematic review software was utilized to identify and remove duplicate records. Subsequently, the screening of titles and abstracts, followed by full‐text evaluation, was conducted independently by the two reviewers. Any persistent discrepancies were resolved through consensus or by a third senior researcher.

### 2.4. Quality Assessment

The methodological quality and reporting integrity of the included systematic reviews were evaluated using the Assessment of Multiple Systematic Reviews 2 (AMSTAR 2) tool. This instrument comprises 16 items that allow for a comprehensive appraisal of systematic reviews of both randomized and nonrandomized studies. Based on the assessment, the reviews were categorized into four levels of confidence: high, moderate, low, or critically low [15].

### 2.5. Data Abstraction

Data from the selected studies were extracted independently by two researchers and managed using Microsoft Excel and Google Docs. The extracted information included bibliographic and sociodemographic data (e.g., author, year, and country); clinical variables related to the amputation (e.g., level of amputation and etiology); pharmacological intervention details (e.g., drug class, dosage, administration route); and pain‐related outcomes (e.g., pain intensity scales for residual and PLP).

### 2.6. Synthesis

A narrative synthesis was first conducted to summarize the characteristics, interventions, and clinical outcomes of all included studies. For the quantitative synthesis, a NMA was performed using a frequentist approach via the “netmeta” package in the R software environment (Version 4.0.3; R Core Team, 2020). Efficacy and safety estimates were calculated using frequencies, means, standard deviations, and 95% confidence intervals (CIs) derived from the primary randomized controlled trials (RCTs).

Prior to the analysis, the network geometry was inspected to determine connectivity. In cases where the network was disconnected, subnetworks were identified and analyzed separately. Subnetworks consisting of only a single study were excluded from the meta‐analysis due to the impossibility of conducting comparative estimates.

## 3. Results

### 3.1. Study Selection

The initial database search yielded 2352 records. After removing duplicates, 841 records remained for title and abstract screening. Following this preliminary phase, 43 articles were selected for full‐text assessment.

**FIGURE 1:**
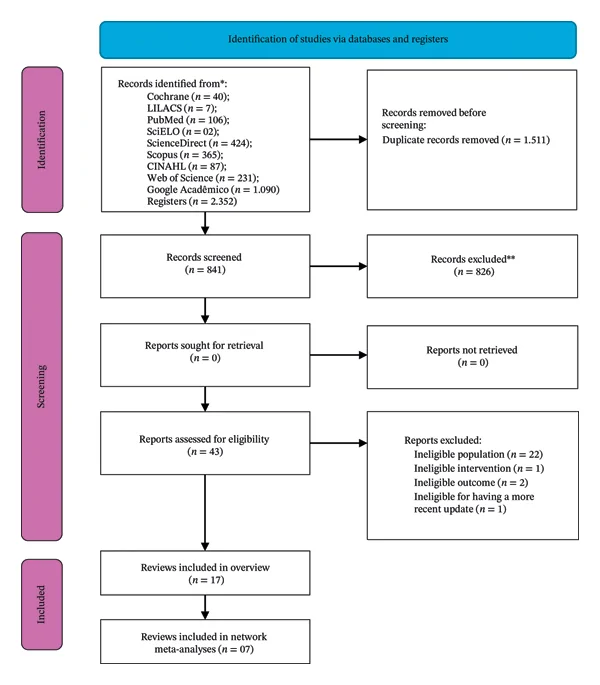
PRISMA 2020 flow diagram for new systematic reviews, which included searches of databases and registers.

Upon comprehensive full‐text review and application of the eligibility criteria, 17 systematic reviews were deemed eligible for inclusion in this study. From this final sample, seven reviews comprising RCTs provided sufficient data for the NMA. The complete process of identification, screening, and selection is detailed in the PRISMA flow diagram (Figure 1).

### 3.2. Synthesis

A summary of the primary characteristics of the 17 included studies is presented in Table 2. Across these studies, a total of 17,011 participants were evaluated, with ages ranging from 19 to 92 years.

**TABLE 2:** Summary of studies. Natal, RN, Brazil, 2025.

Author/year/country/language	Objectives	No. of studies/type of study/follow‐up	Participants/sex/age	Amputation site/cause of amputation	Pain/assessment scales and instruments	Purpose/period of application/type of pain	Terapias	Main results
Alviar et al./2016/Australia/English	To summarize the evidence for the effectiveness of pharmacological interventions in the treatment of phantom limb pain.	14/RS/Ranged from 30 min to 2 years	89/189 men and 80 women/19–81 years old	Upper and lower limbs/traumatic, vascular, neoplastic, infectious, and chronic pain syndromes	NRS, VAS, West Haven‐Yale Multidimensional Pain Inventory.	Treatment/postoperative/phantom limb pain	Study 01: Group 1—botulinum toxin; Group 2—combination of botulinum toxin with lidocaine; Group 3—combination of botulinum toxin with methylprednisolone. Study 02: Group 1—memantine; Group 2—placebo. Study 03: Group 1—memantine; Group 2—placebo. Study 04: Group 1—memantine; Group 2—placebo. Study 05: Group 1—dextromethorphan; Group 2—placebo. Study 06: Group 1—ketamine; Group 2—placebo. Study 07: Group 1—ketamine; Group 2—calcitonin; Group 3—combination of ketamine and calcitonin; Group 4—placebo. Study 08: Group 1—gabapentin; Group 2—placebo. Study 09: Group 1—gabapentin; Group 2—placebo. Study 10: Group 01—amitriptyline; Group 02—benztropine mesylate. Study 11: Group 1—calcitonin; Group 2—placebo. Study 12: Group 1—ketamine; Group 2—calcitonin; Group 3—combination of ketamine and calcitonin; Group 4—placebo. Study 13: Group 01—morphine sulfate; Group 2—placebo. Study 14: Group 1—bupivacaine; Group 2—placebo.	Overall, the evidence of efficacy for the reviewed drugs is so far inconclusive. Adverse effects—memantine: dizziness, tiredness, fatigue, dizziness, headache, nausea, restlessness, excitement, agitation, cramps. Ketamine: drowsiness, nausea, facial flushing, hot/cold, flushing, dizziness, lightheadedness, discomfort, mood elevation, loss of consciousness, sedation, hallucination, hearing impairment, impairment. Gabapentin: drowsiness, dizziness, headache, and nausea. Amitriptyline: dry mouth and dizziness. Calcitonin: headache, dizziness, nausea, vomiting, phantom sensation, drowsiness, hot/cold, flushing. Morphine: constipation, tiredness, dizziness, sweating, difficulty urinating, dizziness, itching, difficulty breathing, sedation.
Jeremy et al./2024/Australia/English	To evaluate the effectiveness of muscle reinnervation aimed at reducing pain and improving functional outcomes for patients who underwent lower limb amputation.	20/RS/ranged from 2 to 22 months	778/NI/34–53 years	Lower limbs/trauma, infection, cancer, ischemia and unspecified causes	Patient‐reported outcomes, NRS.	Prevention/intraoperative/acute pain and phantom limb pain	Targeted muscle reinnervation therapy, primary and secondary. Comparator group of studies not informed.	The therapy significantly reduced both residual and phantom limb pain, decreased opioid dependence, increased ambulation rates, and improved use of prosthetic devices. Adverse effects—recurrent pain from neuromas; early postoperative neurogenic pain; permanently anesthetized areas of skin over the stump and mid‐thigh; susceptibility to ulceration when patients wear a prosthesis.
Eldaly et al./2024/United States/English	To evaluate the effectiveness of virtual reality and augmented reality in reducing phantom limb pain in adult upper and lower limb amputees and adult patients with brachial plexus avulsion injuries.	09/RS/varied from 2 to 12 months	139/NI	Upper and lower limbs/NI	NRS, Pain Rating Index, QDM, QDM Short Form, VAS, Phantom Limb Pain Questionnaire, IMD, Weighted Pain Distribution Scale, Neuropathic Pain Symptom Inventory, IBD.	Treatment/post‐operative/phantom limb pain	Study 01: Group 1—virtual reality; Group 2—augmented reality; Group 3—machine learning; Group 4—serious games. Study 02: Group 1—virtual reality; Group 2—mirror visual feedback. Study 03: Group 1—virtual reality. Study 04: Group 1—integrated virtual environment. Study 05: Group 1—augmented reality; Group 2—traditional mirror therapy. Study 06: Group 1—virtual reality; Group 2—mirror visual feedback. Study 07: Group 1—virtual reality; Group 2—mirror visual feedback. Study 08: Group 1—augmented reality. Study 09: Group 1—virtual reality.	Virtual reality or augmented reality cannot be recommended for the treatment of phantom limb pain. Current literature provides limited evidence on the efficacy of these new therapies. Adverse effects—mild nausea, emotional reaction and increased sweating at the beginning of treatment.
Garcia‐Pallero et al./2020/Spain/English	To analyze the effectiveness of central nervous system stimulation therapies for pain management in people with phantom limb pain.	10/RS/NI	173/NI	Upper and lower limbs/NI	EVA	Treatment/postoperative/phantom limb pain	Central nervous system stimulation therapies. No comparator group for the studies reported.	Transcranial magnetic stimulation may be an effective treatment for minimizing the sensation of phantom limb pain, even 3 or 4 weeks after a single treatment session. The evidence for transcranial direct current stimulation to date is controversial. Adverse effects—not reported.
Hali et al./2024/Canada/English	To summarize current research on the effect of immersive virtual reality in the treatment of phantom limb pain, as determined by patient‐reported scores on objective pain measures.	15/RS/NI	109/97 men and 12 women/average age 55 years	Upper and lower limbs/NI	Short Form of QDM; NRS; EVE; IMD	Treatment/postoperative/phantom limb pain	Virtual reality. No information provided on the comparison group of the studies.	Published literature reports an acute decrease in phantom limb pain after a single virtual reality session, as well as when applied in multiple sessions. Adverse effects—not reported.
Laloo et al./2021/United Kingdom/English	To evaluate the effect of perineural catheters on postoperative pain, postoperative morphine requirement, hospital mortality, long‐term phantom limb pain, and chronic stump pain.	10/RSM/NI	731/NI	Lower limbs/NI	NRS	Treatment/postoperative/chronic stump pain and phantom limb pain	Study 1: General anesthesia. Group 2—not informed. Study 2: Group 1—general anesthesia; Group 2—spinal anesthesia. Study 3: Group 1—general anesthesia; Group 2—epidural anesthesia; Group 2—spinal anesthesia. Study 4: Group 1—general anesthesia; Group 2—epidural anesthesia; Group 2—spinal anesthesia. Study 5: not informed. Study 6: not informed. Study 7: Group 1—general anesthesia; Group 2—spinal anesthesia. Study 8: Group 1—general anesthesia; Group 2—epidural anesthesia; Group 2—spinal anesthesia. Study 9: General anesthesia. Group 2—not informed. Study 10: not informed	Use is associated with reduced pain scores and reduced postoperative morphine requirements. No evidence has been demonstrated for an effect on mortality, phantom limb pain, or chronic stump pain. Adverse effects—not reported.
Limakatso et al./2023/South Africa/English	To critically evaluate relevant data on the effectiveness of graded motor imagery and its components to reduce phantom limb pain and disability in amputees.	11/RSM/NI	373/297 men and 76 women/29 to 62 years old	Upper and lower limbs/NI	EVA, NRS	Treatment/postoperative/phantom limb pain	Study 1: Group 1—mirror therapy; Group 2—control treatments on pain intensity. Study 2: Group 1—mirror therapy; Group 2—covered mirror therapy. Study 3: Group 1—mirror therapy; Group 2—covered mirror therapy. Study 4: Group 1—mirror therapy; Group 2—covered mirror therapy. Study 5: Group 1—graded motor imagery; Group 2—routine care. Study 6: Group 1—graded motor imagery; Group 2—routine care. Study 7: Group 1—mirror therapy; Group 2—control treatments on pain intensity. Study 8: Group 1—mirror therapy; Group 2—control treatments on pain intensity. Study 9: Group 1—mirror therapy; Group 2—mirror therapy with augmented reality. Study 10: Group 1—mirror therapy; Group 2—control treatments on pain intensity. Study 11: Group 1—mirror therapy; Group 2—pain intensity control treatments.	Weak but promising evidence was found that graded motor imagery is more effective than routine treatment in producing clinically significant change in pain severity and that mirror therapy is more effective than mental imagery techniques. Adverse effects—not reported.
Xavier et al./2020/Brazil/Portuguese	To analyze the evidence regarding measures for the control or remission of chronic pain in the stump or phantom limb in adults and elderly people with limb amputation.	04/RS/Varied from 6 to 12 months	160/NI	Upper and lower limbs/peripheral vascular disease, trauma, tumor, infection, and complications of diabetes mellitus	NI	Treatment and prevention/perioperative and postoperative/chronic pain, stump pain, and phantom limb pain	Study 1: Group 1—continuous epidural analgesia in the perioperative period (bupivacaine and fentanyl). Epidural anesthesia (bupivacaine and fentanyl). Group 2—preoperative analgesia with fentanyl; in the peri‐ and postoperative period; continuous epidural analgesia (bupivacaine and fentanyl). Group 3—pre‐ and postoperative analgesia with fentanyl; continuous epidural analgesia (bupivacaine and fentanyl). Group 4—pre‐ and postoperative analgesia with fentanyl. General anesthesia. Group 5—Pre‐ and postoperative analgesia with intramuscular meperidine plus oral codeine/paracetamol. If necessary, additional analgesia with paracetamol plus parecoxib. General anesthesia. Study 2: Group 1—combination of epidural bupivacaine, calcitonin and fentanyl; Group 2—combination of epidural bupivacaine and fentanyl. Study 3: phantom motor execution, with the aid of virtual reality, augmented reality and game. Comparator group not informed. Study 4: Group 1—gradual motor imagery; Group 2—control, conventional physiotherapy.	The findings suggest a protective effect of preventive pharmacological therapies, via the epidural route, in combination with bupivacaine and fentanyl or added to calcitonin, in the perioperative period. Promising data are also presented for nonpharmacological therapies for pain control, phantom motor execution and gradual motor imagery. Adverse effects—not reported.
Dixon et al./2023/United Kingdom/English	To explore the professional medical conversation about the clinical management of chronic postamputation pain in this cohort of patients, the development over the 20th century, and how information was disseminated among medical professionals.	101/RS/NI	9.326/NI	Upper and lower limbs/gunshot wounds, projectile and explosion wounds.	NI	Treatment/postoperative/residual limb pain; stump pain; phantom limb pain; phantom limb sensation; neuropathic pain.	Surgical techniques, physiotherapy, multidisciplinary treatment, and pharmacological interventions (morphine, papaverine, codeine and noscapine, aprobarbital, a bromide called Sedobrol and phenobarbital). No randomized control trials were found; therefore, there are no comparisons.	It is difficult to interpret how successful the treatments in the retrieved texts were in reducing or abolishing chronic postamputation pain, there was no consistency among the reports with little standardized language and none of the standard reporting structures used in medical journals today. Adverse effects—not reported.
Tham et al./2022/United Kingdom/English	To assess data quality and determine the effectiveness of targeted muscle reinnervation for pain reduction and functional outcomes in amputees.	10/RSM/NI	1343/men only/35 to 59 years old	Upper and lower limbs/NI	NRS, PROMIS, EVA	Treatment/postoperative/residual limb pain and phantom limb pain	Targeted muscle Reinnervation. No comparator group for the studies was provided.	The review demonstrated overall improvement in pain and functional outcomes in amputees following therapy compared with standard care or no treatment. Adverse effects—not reported.
Plato et al./2018/Finlândia/inglês	To evaluate the efficacy and safety of analgesic interventions for acute pain after major limb amputation.	19/RS/NI	949/NI/36 and 92 years old	Upper and lower limbs/peripheral vascular disease, malignant disease, trauma	EVA, QDM	Treatment/preoperative, perioperative and postoperative/acute pain, stump pain and phantom limb pain.	Study 1: Group 1—preoperative epidural bupivacaine, fentanyl for 48 h, preoperative and postoperative epidural anesthesia for 48 h; Group 2—preoperative fentanyl for 48 h, epidural anesthesia, postoperative epidural infusion as for 48 h; Group 3—preoperative fentanyl for 48 h, epidural anesthesia, postoperative fentanyl for 48 h; Group 4—preoperative fentanyl for 48 h, general anesthesia, postoperative fentanyl for 48 h; Group 5—Preoperative and postoperative analgesia with pethidine, codeine, paracetamol, and general anesthesia. Study 2: Group 1—epidural bupivacaine, diamorphine 24 h preoperative and 72 h postoperative; Group 2—Continuous sciatica (also known as common tibial or peroneal), nerve block with bupivacaine 72 h postoperatively. Study 3: Group 1—epidural bupivacaine, morphine 18 h preoperatively and median 166 h postoperatively; Group 2—epidural saline, median morphine 18.5 h preoperatively and epidural analgesia with bupivacaine, median morphine 166 h postoperatively. Study 4: Group 1—epidural anesthesia with ketamine and bupivacaine; Group 2—epidural anesthesia with saline and bupivacaine before the start of the operation, continuous infusion of saline and bupivacaine 48–72 h postoperatively. Study 5: Group 1—epidural anesthesia with bupivacaine, fentanyl and calcitonin preoperatively and postoperatively; Group 2—epidural anesthesia with bupivacaine, fentanyl and saline solution preoperatively and postoperatively. Study 6: Group 1—Epidural bupivacaine and morphine 72 h preoperatively; Group 2—various analgesics: paracetamol, NSAIDs, opioids starting 72 h before amputation. Study 7: Group 1—epidural bupivacaine, clonidine, diamorphine preoperatively and 72 h postoperatively; Group 2—opioid analgesia as needed.	It cannot be stated that there are effective and safe ways to treat acute postoperative pain after major amputation without clinically significant side effects and with the benefit of preventing chronic pain. Overall, there appears to be very little data supporting current clinical practice on pain management in acute postamputation pain. Adverse effects—epidural analgesia: nausea, vomiting, sedation, confusion or hallucinations, transient urinary retention, fecal incontinence, meningitis, subcutaneous abscess. Gabapentin: nausea, stomach pain, fatigue, confusion, nightmares. Memantine: nausea, dizziness, headache, and agitation.
Wang et al./2021/China/English	To evaluate the effects of mirror therapy on phantom limb sensation and phantom limb pain in amputees.	11/RSM/NI	491/NI/19 to 61 years old	Upper and lower limbs/Diabetes mellitus, vascular disease, tumor, trauma	EVA, NRS	Treatment/post‐operative/phantom limb pain	Study 1: Group 1—mirror therapy; Group 2: covered mirror. Study 2: Group 1—mirror therapy; Group 2: covered mirror. Study 3: Group 1—mirror therapy; Group 2: covered mirror, mirror therapy; Group 3—mirror therapy and mental imagery. Study 4: Group 1—mirror therapy; Group 2: mental imagery. Study 5: Group 1—mirror therapy; Group 2: transcranial electrical nerve stimulation. Study 6: Group 1—mirror therapy; Group 2: covered mirror and mental imagery. Study 7: Group 1—mirror therapy; Group 2: covered mirror and mirror therapy. Study 8: Group 1—mirror therapy; Group 2: tactile stimulation; Group 3—mirror therapy and tactile stimulation. Study 9: Group 1—mirror therapy; Group 2: sensorimotor exercises and automated sensorimotor exercises. Study 10: Group 1—mirror therapy; Group 2: mirror therapy and mirror treatment; Group 3—mirror therapy and automated sensorimotor exercises. Study 11: Group 1—mirror therapy; Group 2: mental imagery.	It is concluded that there is reasonable quality evidence that mirror therapy is beneficial in reducing phantom limb pain. Adverse effects—worsening of phantom limb sensation and/or phantom limb pain, telescopic distortion of the phantom limb, nausea, dizziness, confusion, feeling irritable and sad.
Xie et al./2022/China/English	To evaluate the effectiveness of mirror therapy for phantom limb pain.	10/RSM/NI	450/NI/28 a 61 anos	Upper and lower limbs/NI	VAS, MDQ, MDQ Short Form, Beck Depression Inventory, Perceived Global Affect Scale, Hamilton Anxiety Scale, Neuropathic Pain Symptom Inventory, German version of the Pain Self‐Efficacy Questionnaire, Pittsburgh Sleep Quality Index Scale, Patient‐Specific Functional Scale, and Universal Pain Score.	Treatment/postoperative/phantom limb pain	Study 1: Group 1—mirror therapy; Group 2: physiotherapy and routine medical care. Study 2: Group 1—mirror therapy; Group 2: covered mirror. Study 3: Group 1—mirror therapy; Group 2: not provided. Study 4: Group 1—mirror therapy; Group 2: transcranial electrical nerve stimulation. Study 5: Group 1—mirror therapy; Group 2: covered mirror; Group 3—mental imagery. Study 6: Group 1—mirror therapy; Group 2: covered mirror. Study 7: Group 1—mirror therapy; Group 2: sensorimotor exercises. Study 8: Group 1—mirror therapy; Group 2: tactile stimulation. Study 9: Group 1—mirror therapy; Group 2: phantom exercises. Study 10: Group 1—mirror therapy; Group 2: mental imagery.	Mirror therapy has beneficial effects for patients with phantom limb pain in the short term. There was no evidence for long‐term use. Adverse effects—not reported.
Mauch et al./2024 Canada/English	To evaluate the effects of targeted muscle reinnervation and primary regenerative peripheral nerve interface at the time of limb amputation on the incidence and intensity of neuroma and postoperative pain.	11/RSM/NI	1.061/NI	Upper or lower limbs/NI	NI	Prevention/intraoperative or postoperative/residual limb pain and phantom limb pain	Study 1: Group 1—primary target muscle reinnervation; Group 2: secondary target muscle reinnervation. Study 2: Group 1—primary target muscle reinnervation; Group 2: secondary target muscle reinnervation. Study 3: Group 1—primary target muscle reinnervation; Group 2: secondary target muscle reinnervation. Study 4: Group 1—primary target muscle reinnervation; Group 2: standard of care. Study 5: Group 1—primary regenerative peripheral nerve interface; Group 2: standard of care. Study 6: Group 1—primary target muscle reinnervation; Group 2: not informed. Study 7: Group 1—primary target muscle reinnervation; Group 2: not informed. Study 8: Group 1—primary target muscle reinnervation; Group 2: standard of care. Study 9: Group 1—primary target muscle reinnervation; Group 2: not informed. Study 10: Group 1—primary target muscle reinnervation; Group 2: standard of care. Study 11: Group 1—primary target muscle reinnervation; Group 2: standard of care.	Overall, observational data suggest that therapies likely reduce the incidence of residual pain and phantom limb pain. Adverse effects—not reported.
Neumüller et al./2023/Canada/English	To comprehensively evaluate the evidence surrounding analgesic effects for patients with phantom limb pain.	06/RS/NI	139/86 men and 53 women/average age 61.8 years	Upper or lower limbs/NI	NI	Treatment/postoperative/phantom limb pain	Study 1: Group 1—bupivacaine, calcitonin and fentanyl; Group 2—bupivacaine and fentanyl. Study 2: Group 1—calcitonin; Group 2—ketamine; Group 3—calcitonin combined with ketamine; Group 4—placebo. Study 3: Group 1—calcitonin; Group 2—placebo. Study 4: Group 1—calcitonin; Group 2—placebo. Study 5: Group 1—calcitonin; Group 2—placebo. Study 6: Group 1—calcitonin; Group 2—placebo.	Current evidence supports the use of calcitonin in the treatment of newly developed phantom limb pain, although the evidence for calcitonin is conflicting in chronic phantom limb pain. Adverse effects—not reported.
Herrador Colmenero et al./2017/Spain/English	To evaluate the effectiveness of mirror therapy, motor imagery, and virtual visual feedback to treat phantom limb pain after amputation.	12/RS/NI	317/NI/19 to 82 years old	upper or lower limbs/NI	Nuclear magnetic resonance imaging (NRS), Phantom Limb Pain Questionnaire, VAS, MDQ, West Haven–Yale Multidimensional Phantom Limb Pain Inventory, Functional Magnetic Resonance Imaging, Universal Pain Score, BDI, Patient Information Form, Mirror Therapy Practice Tracker Booklet, Pain Rating Index, and Weighted Pain Distribution Scale.	Treatment/postoperative/phantom limb pain	Study 1: Group 1—motor imagery; Group 2—routine medical care. Study 2: Group 1—mirror therapy; Group 2—covered mirror. Study 3: Group 1—mirror therapy; Group 2—mental imagery. Study 4: Group 1—body‐scan exercise and imagination of movement and sensation in the phantom limb; Group 2—routine medical care. Study 5: Group 1—mirror therapy; Group 2—mental imagery. Study 6: Group 1—virtual reality for upper limb amputation; Group 2—virtual reality for lower limb amputation. Study 7: Group 1—motor imagery; Group 2—general exercises. Study 8: Group 1—mirror therapy; Group 2—motor imagery. Study 9: Group 1—progressive muscle relaxation, mental imagery and phantom exercises; Group 2—physical therapy. Study 10: Group 1—mirror therapy; Group 2—transcutaneous nerve stimulation. Study 11: Group 1—virtual reality; Group 2—augmented reality. Study 12: Group 1—hands‐on mirror therapy training; Group 2—telephone training for mirror therapy.	All studies reported significant pain reduction regardless of the technique used. However, the evidence for the efficacy of these techniques for reducing phantom limb pain in amputees was classified as limited. Adverse effects—not reported.
Othman et al./2018/New Zealand/English	To assess the evidence of efficacy or effectiveness of nonpharmacological interventions in the treatment of phantom limb pain in adults with lower limb amputation.	04/RS/Varied from 1 to 6 months	204/165 men and 39 women/average age 52.01	upper or lower limbs/vascular disorders and trauma	Prosthesis Assessment Questionnaire, IBD, Leeds Assessment of Neuropathic Symptoms and Signs, Pain Questionnaire, NRS, VAS.	Treatment/postoperative/phantom limb pain	Study 1: Group 1—Combined training of progressive muscle relaxation, mental imagery, and phantom exercises; Group 2—physical therapy. Study 2: Group 1—truly noninvasive limb coverage; Group 2—sham noninvasive limb coverage. Study 3: Group 1—true transcranial magnetic stimulation; Group 2—sham transcranial magnetic stimulation. Study 4: Group 1—mirror therapy; Group 2—mental imagery.	Combined treatments using muscle relaxation, imagery and phantom exercises are superior to other treatments. However, the available literature investigating the efficacy and/or effectiveness of interventions in the management of phantom limb pain in lower limb amputation is scarce, with insufficient quality to determine a treatment value. Adverse effects—not reported.

*Note:* Systematic review with meta‐analysis (SMR), Multidimensional Pain Inventory (MDI). Source: research data, 2025.

Abbreviations: BPI, Brief Pain Inventory; MPQ, McGill Pain Questionnaire; NAS, numeric analog scale; NI, not reported; NRS, numeric rating scale; PROMIS, Patient‐Reported Outcomes Measurement Information System; SR, systematic review; VAS, visual analog scale.

Notably, none of the included studies reported specific data regarding the ethnicity or racial background of the participants. This represents a significant gap in the primary literature concerning the demographic representation of the study populations.

The 17 systematic reviews were published between 2016 and 2024, originating from Canada (3), the United Kingdom (3), Australia (2), Spain (2), China (2), South Africa (1), Brazil (1), the United States (1), Finland (1), and New Zealand (1). Six studies included a meta‐analysis, and seven were composed exclusively of RCTs. The population comprised patients with both upper and lower limb amputations.

Seven primary etiologies for amputation were reported: vascular disorders, neoplasms, trauma, infectious diseases, diabetes mellitus, chronic pain, and penetrating injuries (stab wounds). A total of 29 different instruments were identified for pain assessment. The most frequent were: visual analog scale (VAS): 11 studies (64.7%); numeric rating scale (NRS): 10 studies (58.8%); and McGill Pain Questionnaire (MPQ): 5 studies (29.4%). Notably, four studies (23.5%) did not clearly specify the pain assessment method utilized.

Regarding therapeutic strategies, 58 distinct interventions were identified, categorized as follows: Pharmacological therapies: 25 (43.1%); complementary and integrative practices: 21 (36.2%); standard or routine care: 8 (13.8%); surgical therapies: 6 (10.3%), including perioperative anesthetics and targeted muscle reinnervation (TMR); physiotherapy: 5 (8.6%); and multidisciplinary treatment: 1 (1.8%).

Standard or routine care encompassed a multiprofessional approach involving pharmacological and nonpharmacological interventions, perioperative care, complication prevention, and psychosocial support [16–18].

The primary clinical objective was pain treatment (*n* = 14; 82.3%), followed by prevention (*n* = 2; 11.8%) and a combination of both (*n* = 1; 5.9%). Interventions were most frequently applied in the postoperative period (*n* = 13; 76.5%), while others spanned the intraoperative/postoperative (*n* = 2; 11.8%), strictly intraoperative (*n* = 1; 5.9%), or all surgical phases (*n* = 1; 5.9%).

Regarding pain characteristics, PLP was the most prevalent, with 10 studies (58.8%) focusing exclusively on this condition. Specific dosages and administration routes for pharmacological therapies were not detailed due to significant data heterogeneity and missing information in the primary reports, despite unsuccessful attempts to retrieve these data by contacting the authors.

### 3.3. Methodological Quality Assessment

The AMSTAR 2 assessment present in Table 3 revealed that among the 17 included reviews, 7 (41.2%) were rated as “moderate,” 7 (41.2%) were rated as “critically low,” and 3 (17.6%) were rated as “low” regarding overall confidence in the results.

**TABLE 3:** AMSTAR‐2 assessment. Natal, RN, Brazil, 2025.

References	AMSTAR‐2 domains																
	1	2	3	4	5	6	7	8	9	10	11	12	13	14	15	16	Overall quality
Alviar et al.	Y	Y	Y	Y	Y	Y	Y	Y	Y	Y	NMA	NMA	N	Y	Y	Y	Moderate
Jeremy et al.	Y	N	Y	PY	N	N	N	Y	N	N	NMA	NMA	N	N	NMA	Y	Critically low
Eldaly et al.	Y	N	Y	PY	Y	Y	N	Y	Y	N	NMA	NMA	Y	Y	PY	Y	Low
Garcia‐Pallero et al.	Y	Y	Y	Y	Y	Y	N	PY	N	N	NMA	NMA	N	N	NMA	Y	Critically low
Hali et al.	Y	N	Y	PY	Y	Y	N	Y	N	N	NMA	NMA	N	Y	NMA	Y	Critically low
Laloo et al.	Y	N	Y	Y	Y	Y	N	PY	N	Y	Y	Y	Y	Y	Y	Y	Low
Limakatso et al.	Y	Y	Y	Y	Y	Y	N	PY	Y	N	Y	Y	Y	Y	Y	Y	Moderate
Xavier et al.	Y	Y	Y	Y	Y	Y	N	PY	Y	N	NMA	NMA	Y	Y	NMA	Y	Moderate
Dixon et al.	Y	Y	Y	PY	N	N	N	N	N	N	NMA	NMA	N	N	NMA	Y	Critically low
Tham et al.	Y	Y	Y	Y	Y	Y	N	Y	Y	N	Y	Y	Y	Y	Y	Y	Moderate
Plato et al.	PY	N	Y	PY	Y	Y	N	Y	Y	N	NMA	NMA	Y	Y	NMA	Y	Low
Wang et al.	Y	Y	Y	Y	Y	Y	N	Y	Y	N	Y	Y	Y	Y	Y	Y	Moderate
Xie et al.	Y	Y	Y	Y	Y	Y	N	Y	N	N	Y	Y	N	Y	N	Y	Critically low
Mauch et al.	Y	N	Y	Y	Y	Y	N	Y	Y	N	Y	Y	Y	Y	Y	Y	Critically low
Neumüller et al.	Y	Y	Y	Y	Y	Y	N	PY	Y	N	NMA	NMA	PY	Y	NMA	Y	Moderate
Herrador et al.	PY	N	Y	Y	N	N	N	PY	N	N	NMA	NMA	N	Y	NMA	Y	Critically low
Othman et al.	Y	Y	Y	Y	Y	Y	Y	Y	Y	N	NMA	NMA	Y	Y	NMA	Y	Moderate

*Note:* NMA: no meta‐analysis conducted. Note 2: *AMSTAR Checklist*. (1) Did the research questions and inclusion criteria for the review include the components of PICO? (2) Did the review report contain an explicit statement that the review methods were established prior to conducting the review, and did the report justify any significant deviations from the protocol? (3) Did the review authors explain their selection of study designs for inclusion in the review? (4) Did the review authors use a comprehensive literature search strategy? (5) Did the review authors perform study selection in duplicate? (6) Did the review authors perform data extraction in duplicate? (7) Did the review authors provide a list of excluded studies and justify the exclusions? (8) Did the review authors describe the included studies in adequate detail? (9) Did the review authors use a satisfactory technique for assessing the risk of bias (RoB) in individual studies that were included in the review? (10) Did the review authors report the funding sources for the studies included in the review? (11) If meta‐analysis was performed, did the review authors use appropriate methods for the statistical combination of results? (12) If meta‐analysis was performed, did the review authors assess the potential impact of RoB in individual studies on the results of the meta‐analysis or other evidence synthesis? (13) Did the review authors provide a satisfactory explanation and discussion of any observed heterogeneity in the review results? (14) If they performed a quantitative synthesis, did the review authors conduct an adequate investigation of publication bias (small study bias) and discuss its likely impact on the review results? (15) Did the review authors disclose any potential sources of conflict of interest, including any funding they received to conduct the review?. Fonte: dados da pesquisa, 2025.

Abbreviations: N, no; PY, partial yes; Y, yes.

The included reviews performed poorly across three critical domains of the AMSTAR 2 tool: (1) Protocol Registration (Item 2): Failure to register a protocol before the commencement of the review, increasing the risk of selective reporting bias; (2) Justification for Exclusions (Item 7): Absence of a detailed list of excluded studies with the specific rationales for their removal; and (3) Risk of Bias Integration (Item 13): Inadequate consideration of the Risk of Bias (RoB) of primary studies when interpreting and discussing the synthesis of results.

### 3.4. NMA

Seven systematic reviews of clinical trials were identified, comprising 72 primary studies. Of these, 24 were duplicates and were excluded, leaving 48 studies for full‐text review and screening. Ultimately, 17 primary studies were included in the intervention networks.

Two outcomes were analyzed, forming subgroups: PLP (*n* = 16 studies) and RLP (*n* = 3 studies). For pain assessment, numerical scales with continuous data were used, as other types of scales with qualitative assessments precluded the performance of a meta‐analysis.

The meta‐analysis for each outcome was conducted using forest plots and combinations of direct and indirect comparisons. The variables analyzed in the plots included the total number of participants, the mean value of the outcome, and the corresponding standard deviation, all reported for both groups. The 95% CIs were also considered.

For both PLP and RLP outcomes related to amputations, networks were constructed based on the experimental and control therapy groups within the RCTs. For the PLP outcome, five subnetworks were identified. The studies evaluated the following interventions: transcranial magnetic stimulation (TMS) applied to the motor cortex compared with stimulation applied to the vertex; lidocaine versus botulinum toxin A; and phantom exercises compared with a general exercise program.

**FIGURE 2:**
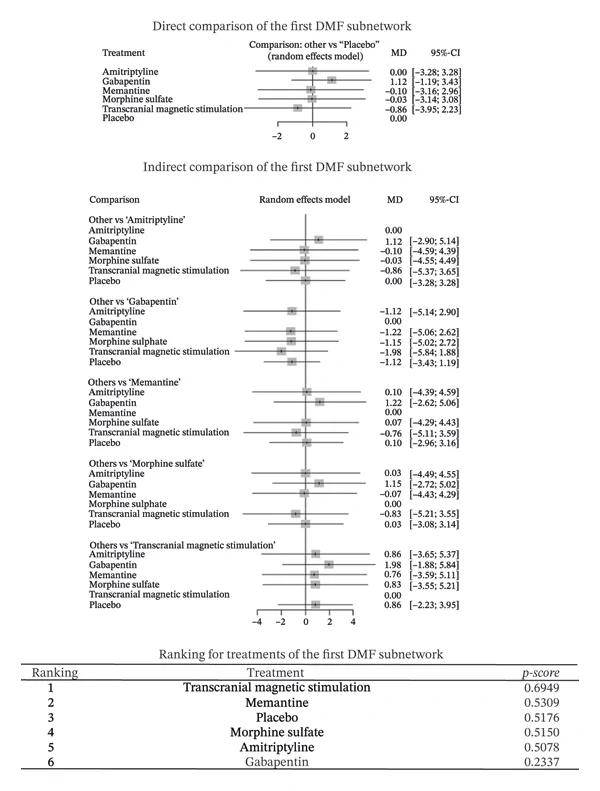
Forest plot comparing each of the treatments (direct and indirect comparison) and ranking of treatments, of the first DMF subnetwork. Natal, RN, Brazil, 2025. *Source:* research data, 2025.

The PLP outcome also allowed for the analysis of two subnetworks. In the first PLP subnetwork, each treatment was compared with placebo, as all studies in this subgroup evaluated therapies (amitriptyline, gabapentin, memantine, morphine, and TMS) against placebo. Figure 2 presents the estimates derived from both direct and indirect comparisons. No statistically significant differences were observed among the therapies, and a treatment ranking is also provided.

Regarding the ranking of therapies for PLP in the first subnetwork, the analysis indicated that the treatment with the highest probability of achieving the best patient outcome was TMS (*p*‐score = 0.6949), followed by memantine (*p*‐score = 0.5309). Gabapentin ranked lowest, with a p‐score of 0.2337.

**FIGURE 3:**
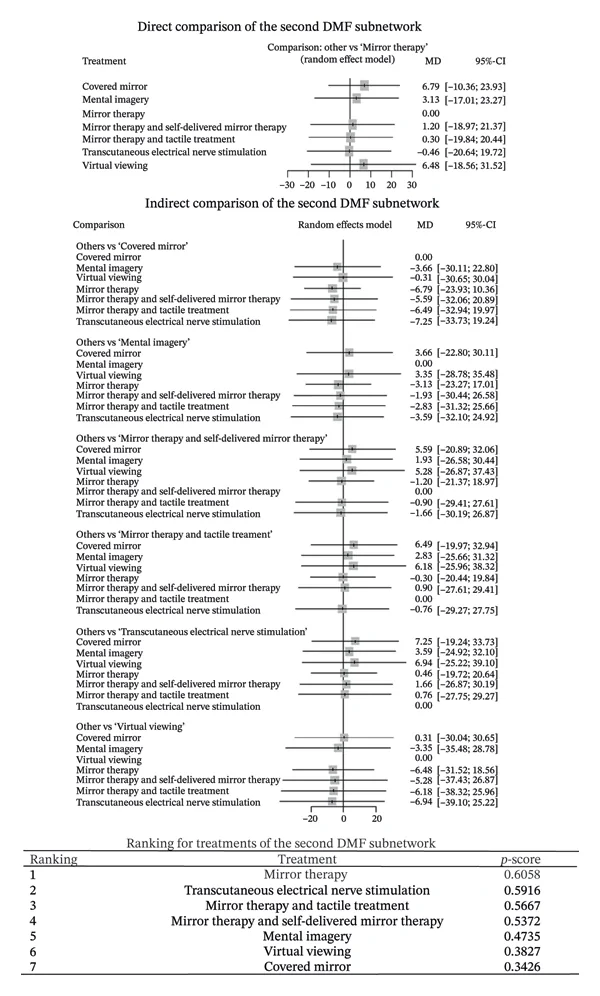
Forest plot comparing each of the treatments (direct and indirect comparisons) and ranking of treatments, of the second DMF subnetwork. Natal, RN, Brazil, 2025. *Source:* research data, 2025.

The adjusted model for the second subnetwork evaluated mirror therapy in comparison with other treatments, as shown in Figure 3. No statistically significant differences were identified in the reduction in PLP among the treatments in either direct or indirect comparisons. According to the treatment ranking for this subnetwork, mirror therapy showed the highest probability of pain reduction (*p*‐score = 0.6058), whereas covered mirror therapy, used as a placebo in the clinical trials, demonstrated the lowest probability (*p*‐score = 0.3426).

For the second outcome, RLP, two networks were identified: one comprising a single study and the other comprising two studies. Consequently, only one subnetwork was suitable for the analysis of the RLP outcome.

**FIGURE 4:**
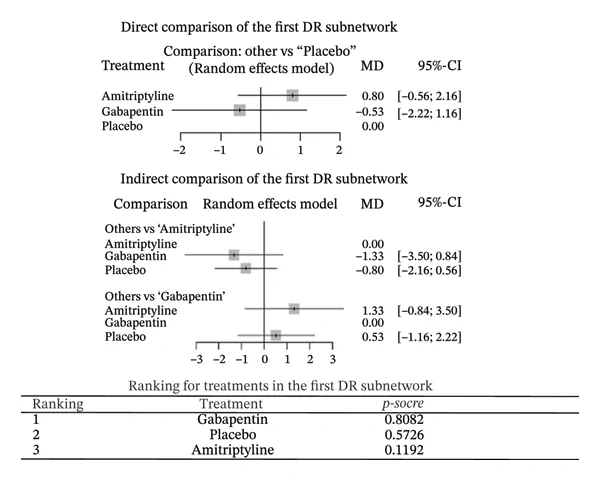
Forest plot comparing each of the treatments (direct and indirect comparison) and ranking of treatments, of the first DR subnetwork. Natal, RN, Brazil, 2025. *Source:* research data, 2025.

The analysis of this RLP subnetwork, which included the pharmacological therapies amitriptyline and gabapentin, did not demonstrate statistically significant differences compared with placebo in either direct or indirect comparisons, as illustrated in Figure 4.

**FIGURE 5:**
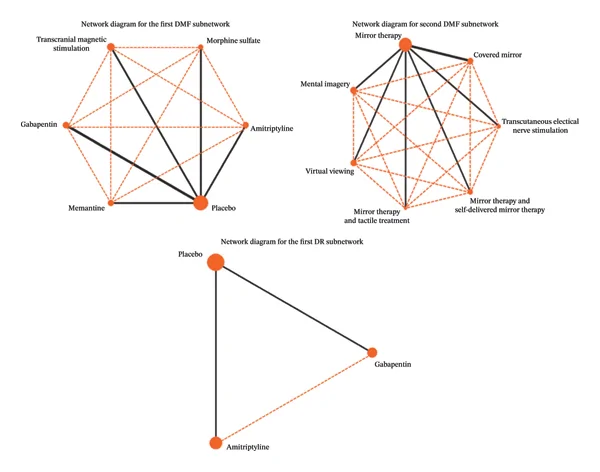
Network diagrams for the DMF and DR subnets. Natal, RN, Brazil, 2025. *Source:* survey data, 2025.

Although no statistically significant differences were detected among the therapies for RLP, the treatment ranking placed gabapentin first (*p*‐score = 0.8082). Finally, well‐connected network diagrams were generated for the PLP and RLP subnetworks, including all interventions, as shown in Figure 5. Solid lines represent the direct comparisons, whereas dashed lines indicate the available indirect comparisons between pairs of interventions.

## 4. Discussion

This study provides a comprehensive evaluation of pharmacological therapies for pain management in postamputation patients, offering insights into their efficacy, safety profiles, and the clinical limitations that impact patient outcomes.

Our findings reinforce that the most affected demographic consists of young and older adult males [19, 20]. Global Burden of Disease data across 204 countries identify a similar trend: men aged 20–60 years are disproportionately affected by amputation due to both clinical conditions and external causes [21]. This gender disparity is partly attributed to higher male representation in physical labor and greater exposure to severe occupational injuries [21]. Furthermore, over 60% of traumatic amputations result from traffic accidents involving young men. Other contributing factors include increased exposure to violence, lifestyle‐related risks, and chronic conditions such as Type 1 diabetes and infectious diseases [19, 21].

In the elderly population, amputation rates increase alongside physiological decline, which elevates the risk of falls, osteoporosis, and the prevalence of metabolic comorbidities [19].

Postamputation pain is a near‐universal experience. Our synthesis confirms that PLP and RLP are the most frequently investigated conditions. While these manifestations are often treated postoperatively, evidence suggests that preventive measures initiated before or during surgery are critical to minimizing symptom intensity.

Effective pain management must begin in the preoperative phase, considering the underlying etiology, the characteristics of pre‐existing pain, and the specific amputation site [22]. Within this context, the nursing team plays a pivotal role. Nurses are often the first to receive patient complaints and are responsible for implementing systematized care plans, leading multidisciplinary actions, and continuously re‐evaluating the effectiveness of interventions [12, 23, 24].

Regarding pharmacological prevention, previous research has highlighted the protective effect of epidural therapies (e.g., bupivacaine combined with fentanyl or calcitonin) administered 48 h prior to the procedure. Patients receiving such preventive measures often report lower pain intensity even when symptoms do manifest [25].

Our review observed that preventive approaches remain underexplored in the literature. Most pharmacological interventions are reactive, initiated in the postoperative period for both RLP and PLP. The identified pharmacological repertoire is vast, including opioids, NMDA antagonists, gabapentinoids (anticonvulsants), antidepressants, and botulinum toxin A, often used in conjunction with nonpharmacological strategies and routine institutional care.

Despite this variety, our NMA revealed a significant lack of statistical superiority among these agents. This suggests that while many drugs are utilized in clinical practice, their comparative efficacy remains unproven, highlighting a critical need for standardized protocols and higher‐quality primary studies.

It is important to note that not all therapies identified in this overview were included in the NMA. To ensure the highest level of evidence and internal validity, only primary RCTs were selected for the quantitative synthesis of therapeutic efficacy.

Opioids and their derivatives including morphine, meperidine, codeine, fentanyl, and pethidine remained the most frequently cited drug class. While opioids are staples in the management of major surgical pain, they present a well‐documented clinical paradox [26]. Despite providing potent acute analgesia, their extensive side‐effect profile including constipation, fatigue, vertigo, urinary retention, pruritus, and life‐threatening respiratory depression significantly compromises patient safety [7].

Furthermore, prolonged opioid use (defined as continuous prescription between 90 and 180 days postamputation) poses severe long‐term risks, such as substance dependence, hyperalgesia, depression, and social withdrawal [12, 24, 26, 27]. These risks are particularly concerning for patients with PLP, a chronic condition that may persist for years. Consequently, developing “opioid‐sparing” strategies remains a critical challenge for the scientific community.

Our findings highlight bupivacaine and fentanyl as the most utilized anesthetics. The literature consistently supports the safety and efficacy of the epidural or perineural approach. Continuous administration via catheters for 72 h postoperatively has been shown to effectively reduce acute pain intensity and may prevent the transition to chronic pain states [6, 25, 28].

Promising alternatives to traditional pharmacotherapy include TMR [18, 29] and nonpharmacological interventions such as mirror therapy, virtual reality, and motor imagery, which were featured in our PLP subnetworks.

Conversely, several pharmacological classes including botulinum toxin A, antidepressants, anticonvulsants, and barbiturates showed limited and often conflicting evidence regarding their efficacy for postamputation pain. Notably, there is currently insufficient evidence to support the routine use of anticholinergics, antispasmodics, or adrenergic agonists, as these lack high‐quality RCTs to justify their clinical administration [6, 9, 30].

Our NMA identified amitriptyline and gabapentin within the RLP and PLP subnetworks. Amitriptyline, a tricyclic antidepressant, modulates serotonin, and norepinephrine levels to assist in pain control. Despite its widespread use for chronic neuropathic pain—common in traumatic amputations, our findings align with previous reports showing no significant superiority over placebo for PLP [7].

Similarly, gabapentin, an anticonvulsant targeting voltage‐gated calcium channels, presented a dichotomous result in our ranking: It placed last for PLP but first for RLP. This discrepancy underscores the controversial nature of gabapentin in this population. Its integration into clinical practice should be approached with caution due to inconsistent efficacy data and a notable profile of adverse effects [7, 9].

Regarding memantine, although it ranked second in our PLP treatment hierarchy, it failed to achieve statistical significance in either direct or indirect comparisons. This supports earlier systematic reviews where daily administration of 30 mg for up to 4 weeks showed no clinically significant reduction in pain intensity [7].

A consistent theme across the reviewed evidence is the shift toward multimodal pain management. Integrating diverse analgesic mechanisms can synergistically enhance anesthetic effects while reducing opioid consumption, hospital stays, and overall healthcare costs.

A successful example of this approach identified in our study is the combination of simple analgesia (e.g., acetaminophen) with anti‐inflammatory agents (e.g., methylprednisolone, parecoxib, or NSAIDs) during the perioperative period [6, 25]. Furthermore, combining pharmacological agents with nonpharmacological therapies—such as mirror therapy or virtual reality represents a promising, holistic frontier for pain management [10, 16, 17, 31].

However, the lack of homogeneity in current study populations limits definitive conclusions. Future RCTs must prioritize standardized protocols to test the long‐term efficacy of these combined therapeutic strategies in the amputee population [32].

Within the PLP intervention networks, two nonpharmacological therapies showed notable ranking probabilities. In the first subnetwork, TMS emerged as the intervention with the highest probability of pain reduction, while mirror therapy led the second subnetwork. Conversely, the lack of studies representing nonpharmacological interventions for RLP identifies a significant gap in the literature and a priority for future research.

TMS facilitates the investigation of neural plasticity and cortical reorganization, providing a framework to modulate maladaptive neuroplasticity and enhance descending inhibitory pathways. When administered repetitively, TMS can adjust cortical excitability, showing promise as a neuromodulatory tool for pain management [33]. However, its therapeutic efficacy for PLP remains inconsistent; while some reviews report improvements in pain intensity, anxiety, and depressive symptoms, others found no significant difference compared to placebo or noted that benefits became negligible after 30 days [34–36].

The primary barriers to establishing the efficacy and safety of TMS for PLP include limited research output, small sample sizes, and a lack of standardized parameters—such as stimulation intensity, session frequency, and pulse count. As observed in current reviews, these technical parameters are crucial for treatment success but remain highly heterogeneous across studies [33–35].

Regarding mirror therapy, five systematic reviews evaluated its application, including variations such as covered mirrors, virtual reality, augmented reality, and gamified exercise programs [10, 16, 17, 31]. Mirror therapy is widely recognized for its clinical feasibility and ease of self‐administration following proper instruction. By reflecting the healthy limb, the therapy re‐establishes a coherent body image, which may alleviate phantom sensations and pain by resolving sensorimotor incongruence [37, 38].

Despite its potential, reviews highlight significant diversity in the implementation of these practices and the instruments used to quantify their effects. This methodological variance hinders the classification of mirror therapy as a definitive “safe and effective” intervention [37, 39]. To overcome this, the development, validation, and dissemination of standardized protocols are essential to ensure replicability in future high‐quality RCTs for the amputee population.

## 5. Limitations

It is essential to acknowledge several limitations that temper the findings of this review. First, there is a profound lack of long‐term longitudinal data; only one included review identified a follow‐up period of up to 2 years in its primary RCTs [6]. In twelve of the seventeen reviews, follow‐up durations were not clearly specified. Consequently, it remains uncertain whether the immediate analgesic benefits and safety profiles of the evaluated therapies persist over time.

Furthermore, the statistical power of certain comparisons was constrained by the fact that several pharmacological agents including botulinum toxin A, dextromethorphan, benztropine mesylate, meperidine, methylprednisolone, and clonidine were evaluated in only a single RCT. This scarcity of primary data precludes robust meta‐analytical synthesis for these specific interventions.

Moreover, the diversity of outcome metrics ranging from various pain scales to imaging findings prevented the inclusion of several potentially relevant studies in the quantitative synthesis, potentially.

Finally, most studies failed to report clinically relevant secondary outcomes, such as quality of life, sleep patterns, or hemodynamic stability (vital signs), which are vital for a holistic understanding of patient recovery. Lastly, as this review focused exclusively on adult and geriatric populations, the findings cannot be generalized to pediatric patients, who may present distinct pathophysiological responses to postamputation pain.

## 6. Conclusions

This systematic review and NMA identified a broad spectrum of pharmacological and nonpharmacological interventions for managing PLP and RLP. Most strategies are reactive, focused on the postoperative period, with preventive approaches remaining significantly underexplored.

The pharmacological landscape is diverse, encompassing opioids, anesthetics, NMDA antagonists, gabapentinoids, and antidepressants, among others. However, all identified pharmacological therapies were associated with adverse effects, posing varying degrees of risk to patient safety. Furthermore, the NMA revealed no statistically significant differences in efficacy across the evaluated therapies in either direct or indirect comparisons. While TMS and mirror therapy achieved the highest relative rankings (P‐scores), their clinical superiority remains unconfirmed due to high study heterogeneity.

Given these results and the identified methodological limitations, current scientific evidence is insufficient to confirm the efficacy and safety of specific pharmacological therapies for pain management in postamputation patients. Many interventions yield inconsistent results, and primary studies often suffer from small, heterogeneous samples and a lack of standardized outcome metrics. Critically, essential aspects of patient well‐being—such as quality of life, sleep patterns, and hemodynamic stability—are rarely addressed in the literature.

Therefore, future research must prioritize methodological rigor and standardized protocols to ensure replicability. Prospective studies should adopt a holistic approach, considering surgical etiology, patient history, socioeconomic factors, and the pharmacokinetics of combined therapies. Specifically, the development of robust, multimodal strategies that integrate pharmacological and nonpharmacological interventions is essential to optimize pain control and enhance the recovery of patients undergoing amputation.

## Funding

This research did not receive any specific grant from any funding agency in the public, commercial, or not‐for‐profit sectors.

## Conflicts of Interest

The authors declare no conflicts of interest.

## Supporting Information

Additional supporting information can be found online in the Supporting Information section.

## Supporting information


**Supporting Information 1** Supporting File 1: Checklist. PRISMA‐NMA (DOC).

**Supporting Information 2** Supporting File 2: Registration in PROSPERO (PDF).

## Data Availability

Data supporting the results of this study are available upon request to the corresponding author.
