# Supplementary material for: Pharmacological Therapies for Pain Management in Patients With Amputation: A Systematic Review and Network Meta‐Analysis
Source: Pain Res Manag. 2026 Jun 4;2026:3654470. doi: 10.1155/prm/3654470 (PMC13581055; doi:10.1155/prm/3654470)
Supplement: Supplementary file 1 [file PRM-2026-3654470-s001.pdf]

NIHR | National Institute for Health and Care Research

PROSPERO
International prospective register of systematic reviews

Drug Therapies for Pain Management in Amputee Patients: Systematic Review and Network Meta-Analysis

Nahadja Tahaynara Barros Leal, Kleyton Santos de Medeiros, Rodrigo Assis Neves Dantas, Daniele Vieira Dantas

To enable PROSPERO to focus on COVID-19 submissions, this registration record has undergone basic automated checks for eligibility and is published exactly as submitted. PROSPERO has never provided peer review, and usual checking by the PROSPERO team does not endorse content. Therefore, automatically published records should be treated as any other PROSPERO registration. Further detail is provided here.

Citation
Nahadja Tahaynara Barros Leal, Kleyton Santos de Medeiros, Rodrigo Assis Neves Dantas, Daniele Vieira Dantas. Drug Therapies for Pain Management in Amputee Patients: Systematic Review and Network Meta-Analysis. PROSPERO 2024 Available from https://www.crd.york.ac.uk/PROSPERO/view/CRD42024500600

REVIEW TITLE AND BASIC DETAILS

Review title
Drug Therapies for Pain Management in Amputee Patients: Systematic Review and Network Meta-Analysis

Original language title
Terapias Medicamentosas para o Manejo da Dor em Pacientes Amputados: Revisão Sistemática e Meta-Análise de Rede

Review objectives
Objective: To evaluate the effectiveness and safety of using drug therapies for pain management in patients undergoing amputation surgery.
Review research question: How effective and safe are drug therapies when compared to the use of placebos and/or other treatments for pain management in amputee patients?
-Population (P): adult and elderly amputees;
-Intervention (I): use of drug therapies;
-Control (C): use of placebos and/or other therapies;

-Outcomes (O): pain management;
-Study design (S): Cochrane Systematic Reviews (CSRs) and non-Cochrane Systematic Reviews (non-CSRs) with or without meta-analysis.

SEARCHING AND SCREENING

Searches

Eligibility criteria:
-The inclusion criteria involved: [1] Cochrane Systematic Reviews (CSRs) and non-Cochrane Systematic Reviews (non-CSRs) with or without meta-analysis. [2] Studies using drug therapies to manage pain in patients aged 18 years or over undergoing amputation surgery.[3] Studies without time or language restrictions.
-The exclusion criteria were as follows: [1] duplicate studies, to be considered only once; [2] studies that do not answer the research question; [3] editorial studies, manuals, dissertations, study protocols, pilot studies, theses, advertising texts, case studies, abstracts published in the annals of scientific events or any type of gray literature that is not characterized as scientific research.
Information sources:
The following databases will be searched: Catálogo de Teses e Dissertações (CAPES), Cochrane Library, Latin American and Caribbean Literature on Health Sciences (LILACS), National Library of Medicine and National Institutes of Health (PubMed), Scientific Electronic Library Online (SciELO), ScienceDirect, Elsevier's Scopus (Scopus), CINAHL, Web of Science and Wiley Online Library e Google Scholar.

Study design

Cochrane Systematic Reviews (CSRs) and non-Cochrane Systematic Reviews (non-CSRs) with or without meta-analysis.

ELIGIBILITY CRITERIA

Condition or domain being studied

Pain management after amputation continues to be a major problem for patients and a challenge for multidisciplinary healthcare teams, as it is a potentially disabling condition, with no consensus on the most effective way to manage this event. Different mechanisms of postoperative pain may develop after nociceptive pain from soft tissue and bone lesions, and neuropathic pain from direct neural trauma and central sensitization. Although acute stump pain improves primarily in the first few postoperative weeks, phantom limb pain (PLP) can occur in up to 80% of amputees, with at least 75% of these patients developing symptoms in the first postoperative week.

Population

Patients over 18 years of age undergoing amputations.

Intervention(s) or exposure(s)

Drug therapies.

Comparator(s) or control(s)

Placebos and/or other therapies.

Context

OUTCOMES TO BE ANALYSED

Main outcomes

Pain management;

Measures of effect

Depends on how the outcome is measured.

Additional outcomes

Quality of life, sleep and Vital Signs (Body Temperature, Pulse Rate, Respiration Rate, Blood Pressure).

Measures of effect

DATA COLLECTION PROCESS

Data extraction (selection and coding)

Registration of studies:
The results will be extracted using the Preferred Reporting Items for Systematic Reviews and Meta-Analyses (PRISMA) flow diagram. Studies of interest will be selected by two researchers (NTBL, KSM), who will carefully read the titles and abstracts independently. Duplicate texts in the databases will be deleted. All selected studies will be evaluated and must meet the inclusion criteria. Discrepancies during selection are resolved by a fifth reviewer (DVD).
Data collection process and management
A standardized data extraction form was developed and tested. Data from each included study will be extracted independently by two reviewers (NTBL and KSM), and any subsequent discrepancies will be resolved through discussion with a third reviewer (DVD). The extracted data will include information about the authors, year of publication, study location, type of study, main objectives, population, pain characterization, efficacy, participant follow-up, pain assessment scales, side effects and quality of life, sleep and vital signs (Body Temperature, Pulse Rate, Respiration Rate, Blood Pressure). Additionally, participant characteristics (e.g., mean age, sex, and cause of amputation) will be collected.

Risk of bias (quality) assessment

Assessment of Multiple Systematic Reviews (AMSTAR 2).

PLANNED DATA SYNTHESIS

Strategy for data synthesis

Data synthesis and analysis
The data will be entered into the R software. This software allows the user to enter protocols; complete reviews; include text, study characteristics, comparison tables and study data; and perform meta-analyses. For dichotomous outcomes, we extracted or calculated the OR and 95% CI for each study. In case of heterogeneity ($I^2 \geq 50\%$), the random effects model will be used

to combine the studies to calculate the OR and 95% CI, using the DerSimonian–Laird algorithm in the meta for package, which provides functions for conducting meta - analyzes in R.
Quality of Evidence
To rate the strength of evidence of the included data, we will use the Grading of Recommendation Assessment, Development, and Evaluation (GRADE) approach. The assessment summary will be incorporated into broader measures to ensure judgment of risk of bias, consistency, openness and accuracy. The quality of the evidence will be assessed based on the risk of bias, indirectness, inconsistency, imprecision and publication bias. Data synthesis and analysis.

Analysis of subgroups or subsets

Subgroup analysis may be carried out depending on the characterization of the pain and the type of amputation performed.

REVIEW AFFILIATION, FUNDING AND PEER REVIEW

Review team members

- Ms Nahadja Tahaynara Barros Leal, Federal University of Rio Grande do Norte
- Professor Kleyton Santos de Medeiros, Liga Contra o Câncer
- Rodrigo Assis Neves Dantas, Federal University of Rio Grande do Norte
- Daniele Vieira Dantas, Federal University of Rio Grande do Norte

Review affiliation

Federal University of Rio Grande do Norte

Funding source

Not Aplicable.

Named contact

Nahadja Tahaynara Barros Leal. nahadja@gmail.com
nahadja@gmail.com

TIMELINE OF THE REVIEW

Review timeline

Start date: 01 June 2024. End date: 01 June 2026

Date of first submission to PROSPERO

27 May 2024

Date of registration in PROSPERO

06 June 2024

CURRENT REVIEW STAGE

Publication of review results

The intention is to publish the review once completed.The review will be published in English

Stage of the review at this submission

Review stage	Started	Completed
Pilot work		
Formal searching/study identification		
Screening search results against inclusion criteria		
Data extraction or receipt of IP		
Risk of bias/quality assessment		
Data synthesis		

Review status

The review is currently planned or ongoing.

ADDITIONAL INFORMATION

PROSPERO version history

- Version 1.1 published on 06 Jun 2024
- Version 1.0 published on 06 Jun 2024

Review conflict of interest

None known

Country

Brazil

Medical Subject Headings

Adult; Aged; Amputation, Surgical; Amputees; Behavior Therapy; Humans; Network Meta-Analysis; Pain Management; Substance-Related Disorders

Disclaimer

The content of this record displays the information provided by the review team. PROSPERO does not peer review registration records or endorse their content.

PROSPERO accepts and posts the information provided in good faith; responsibility for record content rests with the review team. The owner of this record has affirmed that the information provided is truthful and that they understand that deliberate provision of inaccurate information may be construed as scientific misconduct.

PROSPERO does not accept any liability for the content provided in this record or for its use. Readers use the information provided in this record at their own risk.

Any enquiries about the record should be referred to the named review contact
